# Prescriptions and Dispensing

**Published:** 1907-12-07

**Authors:** 


### THERAPEUTICS.

### PRESCRIPTIONS AND DISPENSING.

### VII. MEDICINES IN LIQUID FORM.

Syrup, glycerin, and thick liquids generally are usually the best things to add to a powder that is to be rubbed smooth; in the stiff mixture that results small lumps cannot evade the pestle in the way they would in a thinner liquid.

℞ Pulveris Rhei compositi ... ... ... ʒiss.

Tincturæ Cardamomorum compositæ ... ℥ss.

Aquam Chloroformi ... ... ... ad ℥iij.

Here there is no thick liquid; but the tincture is quite suitable to add to the powder. An alcoholic liquid penetrates better than water into a powder containing ginger, as this does, the oil and resin of which are soluble in alcohol, but not in water.

When any two ingredients are liable to react with one another—and this is a very common case, and a question which must always be considered—the order of mixing should almost always be that which will prevent the reaction, or at least retard it as much as possible. The only exception to this " always " is provided by cases where it is the actual wish of the prescriber that the change should occur. For instance, the following or a similar prescription is sometimes seen:—

℞ Potassii Bicarbonatis ... ... ... ʒj.

Acidi Citrici ... ... ... ... ℈ij.

Syrupi Aurantii ... ... ... ... ℥ss.

Aquam ... ... ... ... ad ℥iv.

Nothing will prevent reaction between the bicarbonate and the citric acid, and it is a very bad prescription unless it is the doctor's desire that his patient should have a solution of potassium citrate saturated with carbonic-acid gas; in dispensing the above the syrup must be added after the effervescence has subsided.

The following are further illustrations of the different results to be obtained by slight differences in the method of mixing:—

℞ Sodii Iodidi ... ... ... ... ... ʒiij.

Tincturæ Nucis Vomicæ ... ... ... ʒij.

Extracti Cinchonæ Liquidi ... ... ʒij.

Glycerini ... ... ... ... ... ʒiv.

Aquam ... ... ... ... ad ℥viij.

If the iodide is dissolved in a little water, the other ingredients added, and the bottle filled up, an unsightly precipitate which cannot be evenly diffused by shaking results from reaction between the sodium iodide and the alkaloids of the cinchona extract. The best plan is to dissolve the iodide in half the water, and add the tincture of nux vomica; mix the liquid extract of cinchona with the glycerin, and add to them the other half of the water. The two liquids may then be mixed together, when the precipitate is formed in a very finely divided state, so that it can be diffused evenly by a gentle shake.

℞ Tincturæ Ferri Perchloridi ... ... ʒj.

Tincturæ Digitalis ... ... ... ... ʒjss.

Acidi Phosphorici diluti ... ... ... ʒjss.

Syrupi Zingiberis ... ... ... ... ℥ss.

Aquam ... ... ... ... ad ℥vj.

If the first four ingredients are put into the bottle without water, or with only a little water, and the latter added afterwards, a very dark mixture results. This is due to the action of the iron on the tannin of the digitalis. If the iron tincture and the phosphoric acid are diluted with half or two-thirds of the water, and the tincture of digitalis and syrup of ginger with the remainder, and the two liquids mixed, there is no such darkening, and this is the method that should be followed.

Considerations such as these lead us on to the general subject of incompatibility, which may be of different kinds. Therapeutic incompatibility, or the prescription together of drugs having opposite actions on the body, is seldom seen, for obvious reasons, though in skilled hands it may occasionally give beneficial results. For example, both tincture of belladonna and tincture of squill may be ordered in cases of cough in children, though the former is a sedative and the latter a stimulating expectorant.

Very many drugs, however, may have a similar therapeutic use, and yet be quite unsuitable for administering together—dispensing incompatibility. An extreme case of this is seen in the following example:—

℞ Misturæ Cretæ ... ... ... ... ℥iij.

Acidi Sulphurici Diluti ... ... ... ʒiij.

Tincturæ Cardamomorum compositæ ... ʒiij.

Aquam ... ... ... ... ad ℥vj.

Chalk mixture is prescribed for diarrhœa, and dilute sulphuric acid also checks the action of the bowels; but, if ordered together in this way, reaction occurs between the calcium carbonate and the acid, carbon dioxide escapes, and calcium sulphate is precipitated.

The problems presented by cases of incompatibility are endless, and require great variety in the methods of dealing with them; only rules of a very general nature can be laid down. The doctor who dispenses for himself will gain experience by his own mistakes. He who prescribes, and gives the prescription to the patient, may accidentally, or through not knowing, write a very bad prescription of the kind under discussion. It is the duty of the chemist to communicate with the doctor before sending out the medicine, if the intentions of the prescription seem to be defeated by the reactions which occur; but it is humiliating to have a prescription referred back in this way. In the following prescription,

℞ Liquoris Arsenicalis ... ... ... ʒij.

Liquoris Strychninæ ... ... ... ʒij.

Ammonii Bromidi ... ... ... ... ʒiij.

Aquam Menthæ Piperitæ ... ... ad ℥vj.

it is clearly the intention of the prescriber to administer both arsenic and strychnine in solution; but if dispensed as written, the alkali of the arsenical solution will decompose the strychnine hydrochloride, and strychnine will be precipitated. This change will probably not occur at once, but only slowly, the strychnine perhaps being deposited as small crystals, with the obvious danger that the patient may receive an overdose of it in the last dose. It is clear that liquor arsenici hydrochloridi should have been prescribed, but it should not be left to the chemist's judgment to make the alteration.

*(To be continued.)*

